# The preparation of polysilicon films on highly boron doped silicon substrates and their effects on Cu out-diffusion

**DOI:** 10.1039/d3ra08772g

**Published:** 2024-02-08

**Authors:** Qingxu Zhang, Zaifu Zhou, Yijun Shen, Weixing Rao, Shihao Xiao, Xiongjie Wu, Lian Zhang, Dandan Liu, Zonghua Wang

**Affiliations:** a College of Chemistry and Chemical Engineering, College of Materials Science and Engineering, Qingdao University Qingdao 266071 China wangzonghua@qdu.edu.cn; b Zhejiang Zhonghe Technology Co., Ltd Hangzhou 310027 China; c Zhejiang Haina Semiconductor Co., Ltd Quzhou 324000 China

## Abstract

The behavior of copper (Cu) diffusion at different storage temperatures of heavily boron-doped silicon substrates is investigated. The surface Cu concentration of the substrate with quantitative Cu contamination exhibits an initial increase followed by a subsequent decrease upon storage at 25 °C and 85 °C. The surface Cu, originating from the out-diffusion, can be effectively removed through RCA cleaning. The polysilicon film, prepared by low-pressure chemical vapor deposition (LPCVD) on the back of the substrate, exhibits a pronounced inhibitory effect on the out-diffusion of Cu. This phenomenon can be attributed to the effective gettering of Cu by both grain boundaries and disordered grain structures within the polycrystalline silicon film. Additionally, the multilayered structure of the polysilicon film exhibits enhanced gettering capabilities. The enhanced gettering effectiveness achieved by the multilayer polysilicon film can be attributed to an increased number of interfaces between layers.

## Abstract 2

The impurity gettering efficiency of the polysilicon film significantly hinders the out diffusion of Cu in the heavily boron-doped mono-silicon substrate. Moreover, as the thickness and layer count of the polysilicon film increase, its gettering effectiveness is further enhanced.

## Graphical abstract

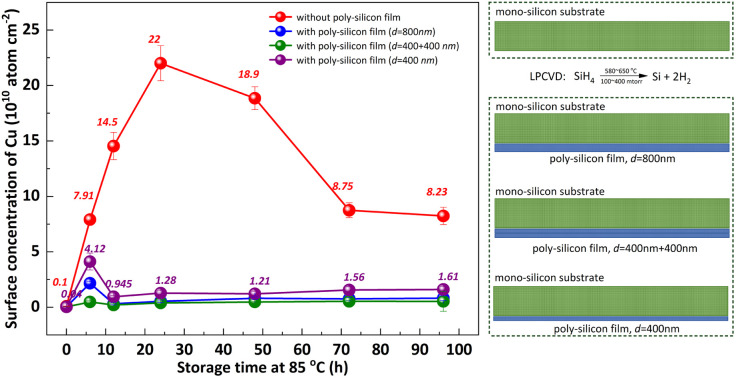

## 1. Introduction

With the development of the microelectronics industry, the ultra-large-scale integrated circuits (ULSI) requirements for silicon materials are increasing.^1^ Currently, p+ silicon substrates doped with highly concentrated boron serve as epitaxial substrates and play a critical role in the fabrication of advanced integrated circuits such as microprocessors, high-value logic devices, and insulated gate bipolar transistors (IGBTs).^2^ The utilization of p+ epitaxy for complementary metal-oxide semiconductor logic devices can effectively mitigate noise in both analog and digital circuits.^3,4^ However, transitional metallic contamination in p+ silicon substrates severely degrades the electrical properties of substrates, and further causes the device failure. On the one hand, metal impurities cannot be completely avoided, due to the purity of the initial material, and the subsequent processing steps.^5^ On the other hand, the increasing performance of electronic devices also places stringent requriements on the concentration of metal contamination. Metallic contamination is specified less than 10^10^ atom per cm^2^ in advanced ULSI technologies. However, contamination up to 10^12^ atom per cm^2^ has been observed during processing.^6^ Among all the metallic contaminants, copper (Cu) is recognized to be the most problematic one. Cu is not only easily transferred to substrates from optimized process tools and low-quality gases and chemicals, but also greatly reduces the yield of silicon devices.^3^ Contamination of Cu can degrade the dielectric properties of gate oxides causing premature breakdown and diffusion into the bulk of the silicon material resulting in an increase of the junction leakage and a reduction of the minority carrier lifetime.^7^

Bulk Cu contamination in the p+ substrates has caused increasing concern in the semiconductor industry of past few decades.^8^ However, both detection and removal of bulk Cu impurities in the p+ silicon substrates are complicated due to the possibility of interaction of Cu with boron.^5^ Cu diffuses in silicon in a positively charged state, which means that its diffusion is also influenced by the doping type and concentration.^9^ In p-type silicon, slow diffusion boron atoms “capture” the quickly diffusible Cu atoms by the covalent interaction, resulting in a lower diffusion coefficient of Cu.^5,10^ Therefore, examination of Cu in silicon and explore effective alternative method to control the Cu concentration in silicon substrates is complex and significant.

Various studies have been conducted on gettering techniques to remove metal impurities from the active region of devices. Gettering techniques using oxide precipitates, polysilicon back seal, ion implantation, and shallow dopants in silicon have been developed over the past few decades.^4^ In particular, polysilicon gettering, as an out gettering method, can form the metal precipitates to removal the metal impurities. The method can effectively inhibit the diffusion of contaminated metals and enhance its gettering effectiveness,^11^ which is resulted from the grain boundaries and highly disordered lattice structure.^12^ The polysilicon films can be obtained by a variety of chemical vapor deposition techniques, including low pressure (LPCVD), atmospheric pressure (APCVD), and plasma enhanced (PECVD). LPCVD, as it affords high deposition volumes, has been studied over the past few decades for numerous electronic applications.^13^ However, there exists a significant disadvantage of warpage increase during LPCVD which affects the subsequent epitaxial growth and chemical mechanical polishing. The warpage increase of the silicon substrates is caused by the residual stress within the polysilicon film.^14^ The removal of residual stresses of polysilicon film is essential, in order to achieve optimum device performance.^15^

Issues of Cu contamination in silicon has not been fully investigated.^16,17^ Bulk Cu contamination in p+ silicon substrates during the substrates manufacturing process, that consists of slicing, lapping, etching, grinding, and polishing, has been rarely studied or reported.^5^ Previous studies mainly focused on the effect of thermal treatment temperature at 200–600 °C on the diffusion of Cu in silicon, while the diffusion of Cu at relatively low temperatures is more instructive for the storage and transportation processes in actual production. In this study, reveal the out-diffusion of Cu in p+ silicon substrates at 25 and 85 °C. The impact of deposition temperature, silane flow rate, and deposition time on the thickness, uniformity, and warpage of polysilicon films was investigated. Finally, by preparing polysilicon film on the backside of p+ silicon substrates, and their inhibition effect on out-diffusion of Cu is systematically studied, figuring out the solution for the Cu contamination in p+ silicon substrates.

## 2. Experimental

### 2.1 Substrates

The heavily boron-doped silicon substrates used in this study are adjacent, as-sawn, sister substrates taken from a high-performance monocrystalline silicon ingot. The concentration of bulk Cu is less than 5.0 × 10^12^ atoms per cm^3^. The monocrystalline silicon substrates were processed according to the standard process of cutting, grinding, acid etching, back treatment, polishing, and post-polish cleaning procedures. The thickness of heavily boron-doped silicon substrate is 625 μm after all the treatment. For cleaning, all the substrates were thoroughly rinsed through RCA cleaning processes to remove surface metals from the substrate as much as possible.

### 2.2 Pre-treatment of Cu contamination

In order to investigate the effect on Cu diffusion, the p+ substrates were pretreated for Cu contamination according to the reported literature.^18^ Briefly, CZ, a p-type substrate with a diameter of 150 mm, with medium oxygen content was chosen. The substrate has a 〈111〉 crystal plane and a resistance of 0.002 Ω cm. The CZ substrates were contaminated from CuCl_2_ standard solution. The resulting surface contamination was in the range of 5.0 × 10^9^ to 5.0 × 10^13^ atom per cm^2^.

### 2.3 Growth of polysilicon film

Polysilicon film was deposited on p+ substrate covered with 500 nm thermal grown SiO_2_ film in a THERMCO 5200 LPCVD furnace. Pure silane was used as the reactant. The furnace tube consists of three independently controlled temperature zones (*T*_1_, bottom of furnace; *T*_2_, middle of furnace; *T*_3_, inlet of furnace) and two independently controlled silane flowmeters (*F*_1_, bottom of furnace; *F*_2_, inlet of furnace). The deposition temperature (*T*_d_) varied from 600 to 660 °C. The pressure 0.3 Torr was used for each procedure. The range of flow rates for the silane was between 130 to 180 sccm. The obtained polysilicon films were available in thickness ranging from 400 nm to 1800 nm. The multilayer polysilicon film was grown several times under different *T*_d_ and deposition time. For multilayer depositions, the silane flow was shut off following deposition of each individual layer and the reactor was allowed to stabilize at the new *T*_d_ before the silane was reintroduced. Each temperature change took 30 min. The monolayer of same thickness would take about 4.5 h at 620 °C and 4.0 h at 650 °C, respectively.

### 2.4 Effects of polysilicon film on Cu diffusion

Thicknesses and uniformity of polysilicon films were measured by an optical thickness instrument (F50 Filmetrics) which utilizes a five-point test method. The geometric parameters were measured by a substrates surface flatness measuring instrument (ADE 7200). The microstructures were determined using ultra-high resolution field emission scanning electron microscope (SEM, Hitachi SU8000) and atomic force microscope (AFM). The precipitation of these metals on the substrate surface was monitored by inductively coupled plasma mass spectrometry (ICP-MS, Agilent 7900). HNO_3_ (69%), HF (49%) and H_2_O_2_ (31%) were used for sample pretreatment as well as for the preparation of the blank and standard solutions for ICP-MS analysis.

## 3. Results and discussion

### 3.1 Out-diffusion of Cu in p+ substrates

**Fig. 1:**
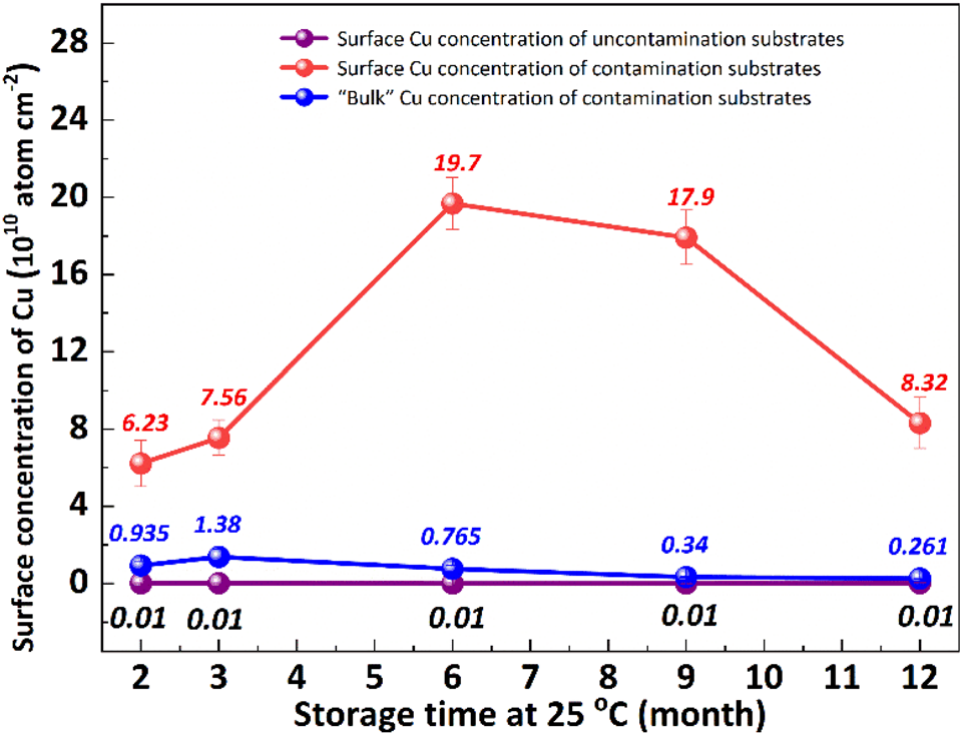
Variations in Cu concentration on p+ substrate front surfaces after storage at 25 °C for different periods of time. The “contaminated substrates” represents the substrates treated by CuCl_2_ for 30 min. The “bulk Cu concentration” represents the surface Cu concentration of substrate after by RCA cleaning and thermal treatment at 300 °C for 2 h.

It is well known that the out-diffusion of Cu in silicon is affected by temperature, doping type, doping concentration, and silicon lattice integrity.^5^Fig. 1 shows the Cu concentration on the front surfaces of substrates after the storage at room temperature (25 °C) for 2, 3, 6, 9, 12 months. All the substrates were cleaned by RCA cleaning before being placed at room temperature to ensure that the surface Cu concentration was below 0.02 × 10^10^ atoms per cm^2^. However, the Cu concentration after thermal treatment at 300 °C for 2 h increased to about 20.0 × 10^10^ atoms per cm^2^, indicating that the Cu contaminated on the surface diffused into the bulk.^18^ In the contaminated substrates, the concentration of surface Cu first increased, then decreased, reaching its maximum after 6 months. This increase in the front surface Cu concentration by 2–3 orders of magnitude indicated that bulk Cu out-diffusion occurred during the room temperature storage. By contrast, there was no significant increase in surface Cu concentration for uncontaminated substrates. This is since the out-diffusion rate is greater than the in-diffusion rate in the early stage, while the opposite trend in the later stage. Then the surface of the same substrate was cleaned by RCA cleaning, and the Cu concentration on the surface after thermal treatment at 300 °C for 2 h was examined as shown in Fig. 1 by the blue symbol, which represents the concentration of bulk Cu. The observed concentration is significantly lower than the initial bulk Cu concentration, indicating that there is a diffusion of Cu from the bulk towards the surface.

**Fig. 2:**
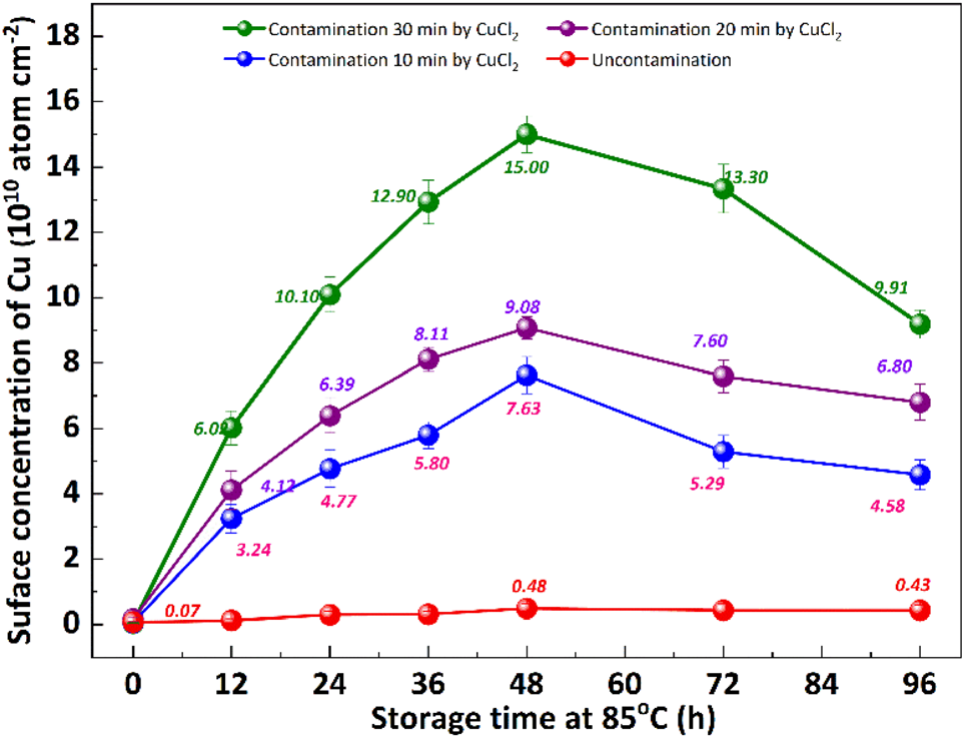
Variations in front surfaces Cu concentration with storage time at 85 °C of p+ silicon substrates after contamination treatment. Three kinds of substrates with different amounts of Cu contamination were treated with CuCl_2_ for 30 min, 20 min, 10 min respectively, and the uncontaminated substrates were not treated by CuCl_2_.

Low-temperature out-diffusion is currently the most commonly used method for monitoring of bulk Cu contamination in high-volume manufacturing.^19^ The out-diffusion mechanism of Cu under various conditions is also explored within a temperature range of 100–400 °C. Cu out-diffusion is predominantly influenced by two key factors, the diffusion coefficient and the solubility of Cu in the silicon matrix.^18^ In principle, the advantageous scenario for Cu out-diffusion involves a high diffusion coefficient coupled with low bulk Cu solubility. However, it is important to note that both of these parameters increase with temperature. Preliminary study results suggest that a thermal treatment temperature around 125 °C is considered optimal for bulk Cu out-diffusion. Around this temperature, Cu out-diffusion is mainly driven by a low bulk Cu solubility and meanwhile Cu diffusivity is sufficient. In addition, the out-diffusion can be further improved by two repeated thermal treatment cycles at 125 °C for 2 h.^18^ However, the diffusion of Cu at relatively low temperature, which is more closed to production temperature, has not been extensively studied. Fig. 2 shows the change of surface Cu concentration at 85 °C. RCA cleaning method was performed on all samples before thermal treatment at 85 °C to ensure the surface Cu concentration was less than 0.02 × 10^10^ atoms per cm^2^. These samples were packaged and sealed separately in incubator. After placing them in an incubator with a constant temperature of 85 °C, the surface Cu concentration was detected at different thermal treatment time, illustrated in Fig. 2. As with the trend at 25 °C (shown in Fig. 1), the Cu concentration in silicon substrates contaminated by CuCl_2_ increased at first and then decreased, reaching its peak after 48 hours. As well, the surface Cu of the uncontaminated samples remained low after 120 h of placement and did not show an increasing trend. Based on the results presented above, it is evident that thermal treatment at 85 °C can facilitate Cu out-diffusion. Also, increasing the temperature and prolonging the period result in a greater rate of Cu out-diffusion.^3^

**Fig. 3:**
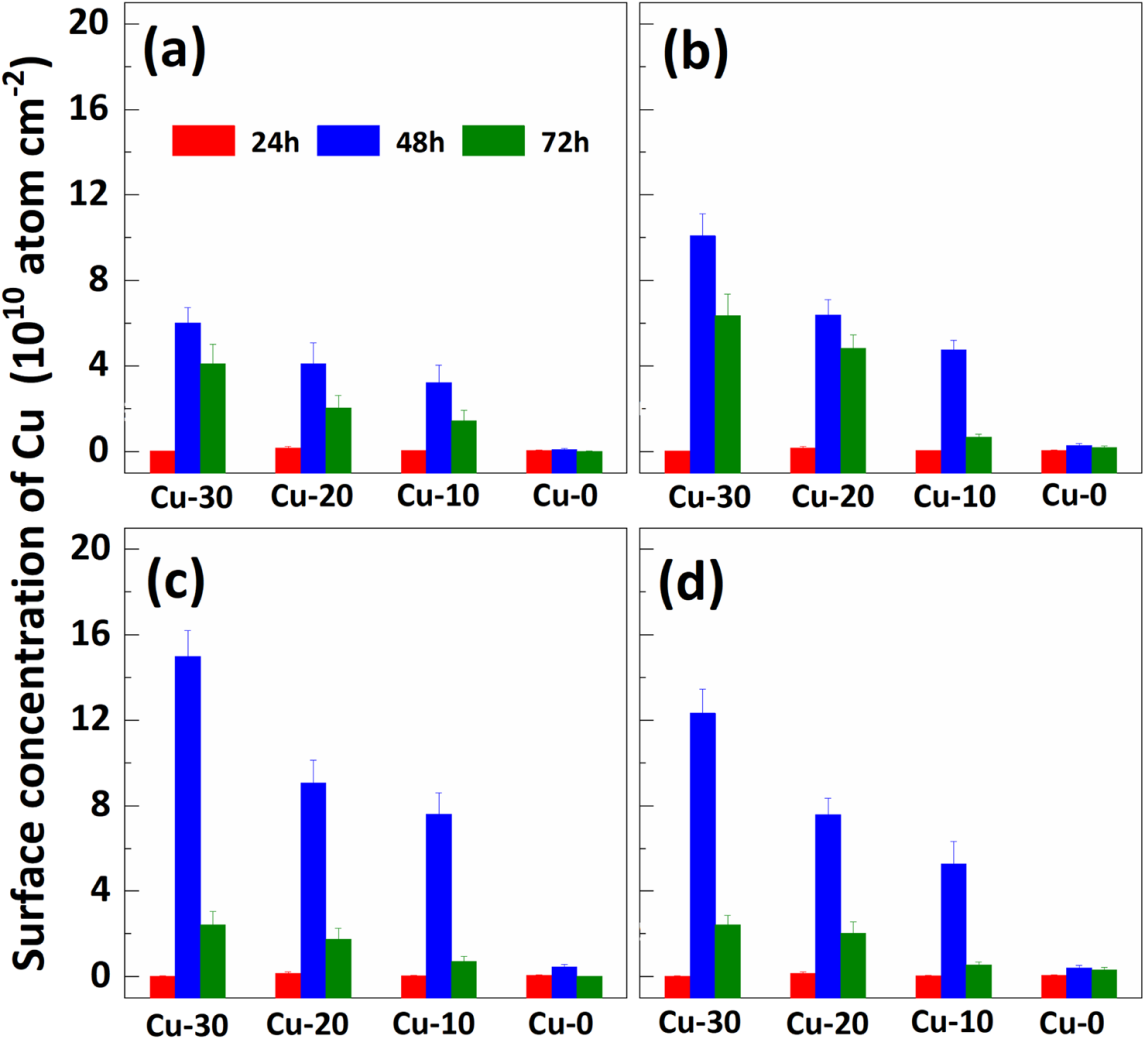
Effect of re-cleaning on surface Cu concentration of p+ substrates with different thermal treatment times. The (a), (b), (c) and (d) correspond to the “first thermal treatment” time of 12, 24, 48 and 72 hours respectively. The 24 h, 48 h and 72 h in (a) represent the time of the “second thermal treatment”, respectively. The Cu-30, Cu-20, Cu-10, and Cu-0 represent the substrates with different amounts of contamination in Fig. 2 respectively.

The impact of re-cleaning on surface Cu concentration of p+ substrates with thermal treatment is depicted in Fig. 3. After the initial 85 °C thermal treatment (as shown in Fig. 2), the surface Cu concentration increases. Subsequently, following the application of the RCA cleaning method (depicted by red column), the surface Cu concentration returns to its initial level, remaining below 0.02 × 10^10^ atoms per cm^2^. However, there is a subsequent increase in the surface Cu concentration after the second thermal treatment at 85 °C for 48 h (illustrated by blue column), albeit at a value lower than that observed after the first thermal treatment. The substrate subjected to an initial heat treatment of 48 hours exhibits the lowest concentration of Cu on its front surface during the subsequent heat treatment. The findings suggest that a significant amount of Cu out-diffusion and thorough RCA cleaning can effectively eliminate the bulk Cu contamination. In other words, Cu out-diffusion can be described as “limited source diffusion”, and the contaminated Cu in the bulk can be effectively eliminated by accelerating its out-diffusion followed by thorough RCA cleaning. It also suggests that the outer diffusion to the surface is not in the form of copper–silicon compounds, but may instead form relatively weak chemical bonds with surrounding atoms.^20^

### 3.2 Deposition polysilicon film on the p+ substrates

**Fig. 4:**
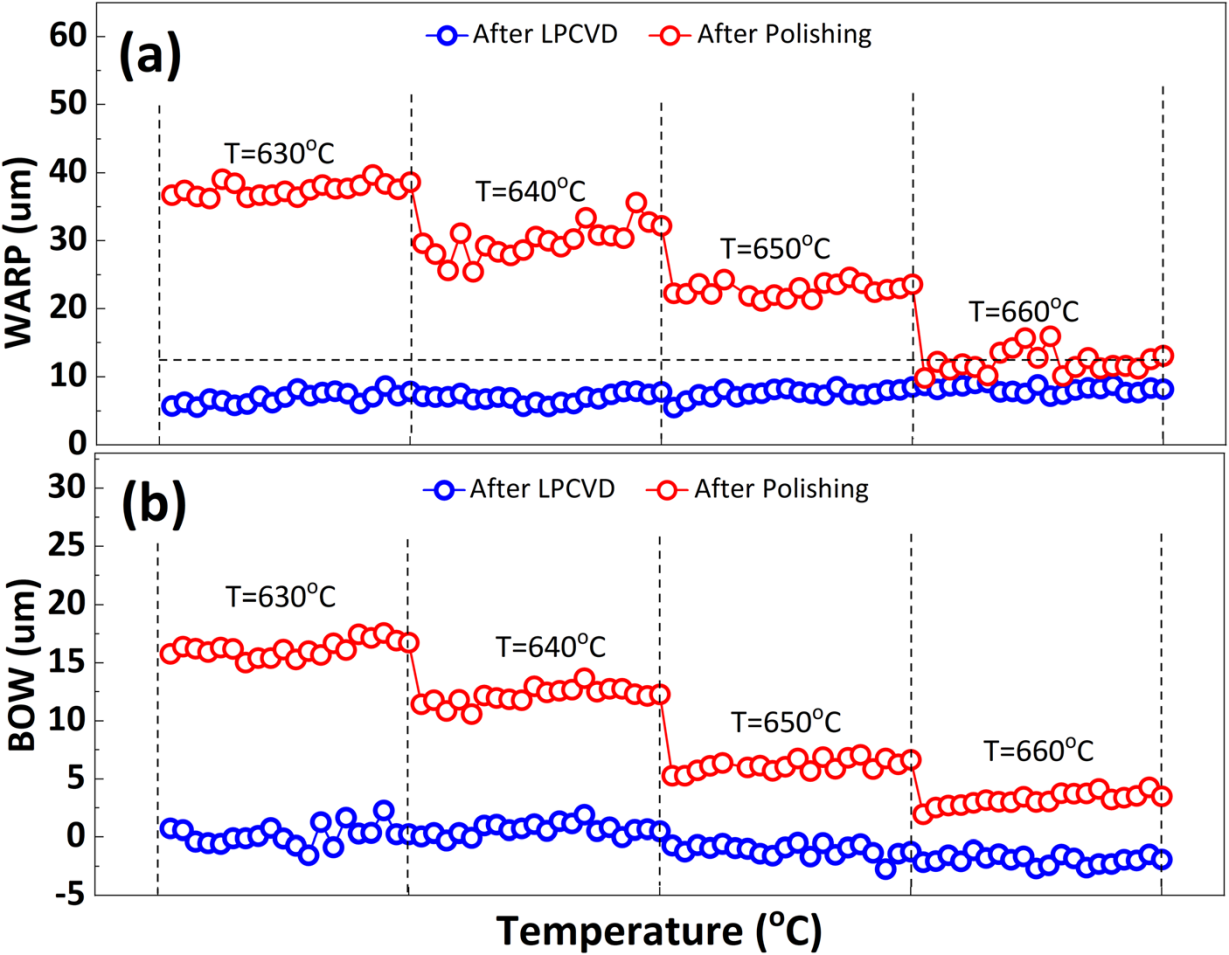
Effect of *T*_d_ on (a) warpage (WARP) and (b) curvature (BOW) of polysilicon film back seal substrates, using silane at a flow rate of 150 sccm and a pressure of 0.3 Torr. All the polysilicon film thickness is about 800 nm by using different deposition times. “After LPCVD” represents that both sides of the substrates have the same thickness of polysilicon film. “After Polishing” represents that the polysilicon film in front surface of the substrates was removed by polishing. Twenty substrates were processed at each *T*_d_.

In order to inhibit the Cu out-diffusion and increase the gettering ability of substrates, a more effective method is to grow a thin film of polysilicon on the backside of the substrates.^21^ Generally, the growth of polysilicon films causes an increase in the warpage of silicon substrates due to the presence of residual stress in film which depending on the deposition temperature (*T*_d_) and silane pressure.^22^Fig. 4 exhibits the influence of the *T*_d_ on warpage in p+ substrates. The polysilicon film was grown on the substrates with a thickness of 800 nm by systematically varying the *T*_d_, silane flow, and deposition time. Subsequently, the warpage (WARP) and curvature (BOW) of the substrates were measured by ADE after one side of the film was removed by polishing. When the same thickness of polysilicon film is deposited on both sides of the substrates, the internal stress on both sides of the film cancels each other, and the result reflects the original warpage and curvature of the substrates. After the one side polysilicon film was removed by polishing, the result reflects the effect of the internal stress of film on the warpage and curvature of the substrates. Based on the findings depicted in Fig. 4, an increase in *T*_d_ leads to a gradual reduction in warpage. Notably, specimens fabricated at 660 °C demonstrate an optimal equilibrium between WARP and BOW, exhibiting a WARP value of approximately 10 and a BOW value of 3. The polysilicon film was positioned beneath the substrate during ADE testing, and the results indicated that there was a tensile stress in the film as evidenced by BOW >0.^14^ Furthermore, it was observed that the magnitude of tensile stress decreased with increasing temperature.

**Fig. 5:**
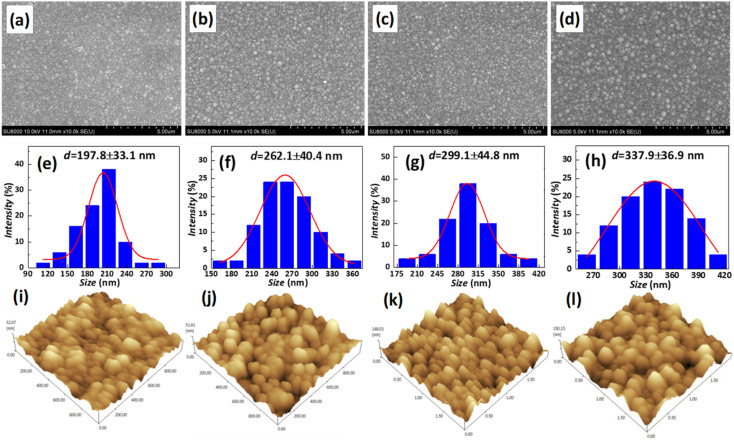
Effect of *T*_d_ on polysilicon grain size. The SEM micrographs in (a), (b), (c), and (d) depict the deposits observed at temperatures of 630 °C, 640 °C, 650 °C, and 660 °C respectively. (e), (f), (g), and (h) depict the particle size distribution statistics based on variables (a), (b), (c), and (d), respectively. The AFM micrographs in (i), (j), (k), and (l) depict the deposits observed at temperatures of 630 °C, 640 °C, 650 °C, and 660 °C respectively. The silane flow rate was set to 180 sccm, while maintaining a pressure of 0.3 Torr for each deposition. The variable in the process were the deposition time and *T*_d_.

Residual stress in a deposition film depends upon properties of film material (such as, thermal expansion coefficient and microstructure of the film) and deposition conditions (such as *T*_d_, pressure, gas flow rate, cooling rate, thermal treatment temperature, and holding time *etc.*). The grain size of polysilicon has a decisive effect on the internal stress of the film.^23,24^ In this study the grain size of polysilicon was observed by SEM and AFM, shown in Fig. 5. According to statistics, the grain size increases with an increase in *T*_d_. The average grain sizes of polysilicon deposited at 630 °C, 640 °C, 650 °C, 660 °C is 197.8 nm, 262.1 nm, 299.1 nm, 337.9 nm, respectively. It is believed that larger grains have smaller internal stresses, as a result their warpage becomes smaller.^25^ According to the analysis of stress theory, the residual stress of polysilicon film is closely related to its microstructure, which is highly dependent on deposition conditions. The slow deposition rate at low temperatures and the limited kinetic energy of silicon atoms contribute to nucleation primarily occurring at the fine grain boundaries, resulting in weakened bonding between the film and substrate, consequently leading to tensile stress.^24^ With the increase in *T*_d_, the kinetic energy of silicon atoms escalates, thereby facilitating the formation and agglomeration of islands, leading to the emergence of larger crystal nuclei. Consequently, this results in a reduction in volume and an increase in stress, ultimately leading to compressive stress. As temperature continues to rise, surface energy and grain size experience growth while atom diffusion occurs between grain boundaries, resulting in a decrease in tensile stress.^14^

**Fig. 6:**
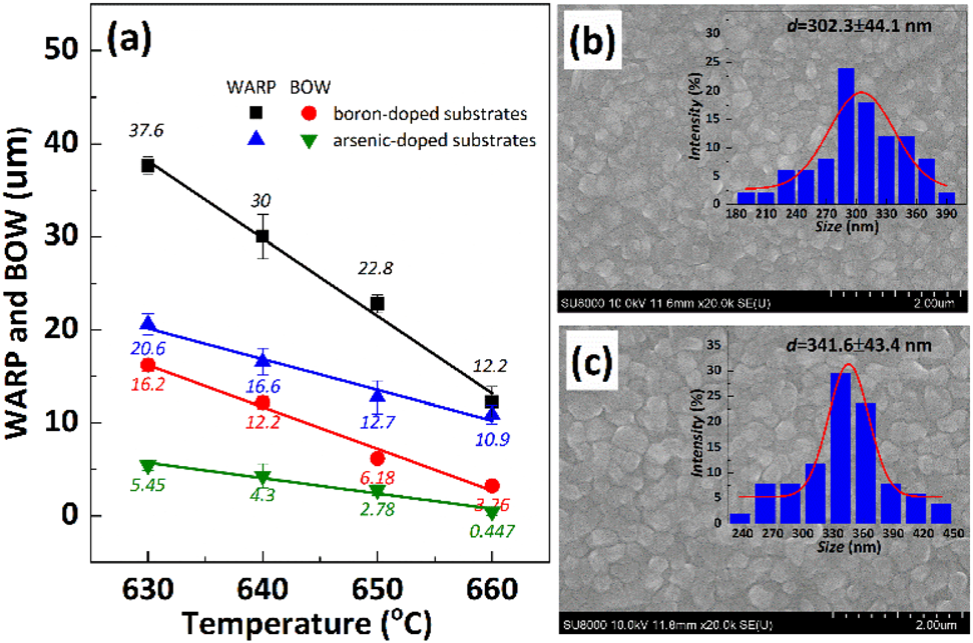
(a) The warpage and curvature of boron-doped and arsenic-doped substrates vary as a function of *T*_d_. (b) and (c) Illustrate the polysilicon grain size of the arsenic-doped substrate at *T*_d_ of 630 °C and 650 °C, respectively. The silane flow rate was set to 180 sccm, while maintaining a pressure of 0.3 Torr for each deposition. The only variable in the process was the *T*_d_.

It is important to note that the internal stress of growing polysilicon films is not only affected by the deposition process, but also by the properties of the substrates, such as doping type, doping amount, crystal orientation, *etc.*, which will affect the warpage of the silicon substrates after the polysilicon film is grown.^25^ Herein, arsenic-doped silicon substrates and boron-doped silicon substrates were deposited with polysilicon at the same *T*_d_ to study the dopant contribute to warpage. The results depicted in Fig. 6(a) demonstrate a significant disparity in compressive stress between the film grown on boron-doped substrates and that grown on arsenic-doped substrates under identical deposition processes. Fig. 6(b) and (c) performs the surface morphology of polysilicon films and their statistical analysis of grain size, to assess the influence of substrates dopants on warpage and curvature. It appears that arsenic-doped substrates exhibit a greater grain size (302 nm and 341 nm) than boron doped substrates (197 nm and 299 nm, as shown in Fig. 5) at the same *T*_d_ (630 °C and 650 °C). There are few studies on the effect of substrates difference on the residual stress of deposited polysilicon films. The reason may be related to surface lattice misalignment caused by different dopants, larger atomic mismatch and slower grain growth associated with boron doped substrates.^26^ The possible explanation is that during the high-temperature deposition process, a significant amount of boron atoms from the heavily doped substrate diffuse into the interior of the polysilicon film. Considering the atomic radii of boron (0.087 nm) and arsenic (0.114 nm), compared to silicon's radius of 0.112 nm, there is a significant mismatch between boron and silicon (0.22), while the mismatch between arsenic and silicon is relatively small (0.02). Therefore, the atomic distortion in the polysilicon film doped with a boron substrate is greater than that in the polysilicon film doped with an arsenic substrate, thereby resulting in an increase in stress within the polysilicon film.^25,27^

**Fig. 7:**
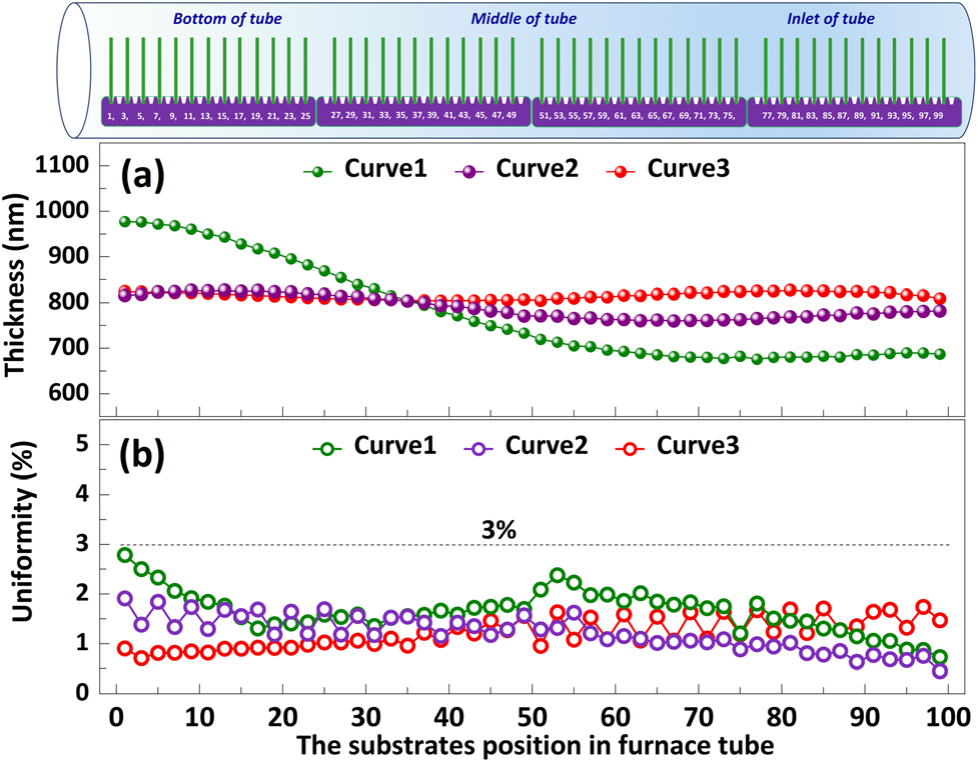
Polysilicon film uniformity and thickness in high-volume manufacturing. The *T*_d_ for LPCVD are designated as *T*_1_ (bottom of the furnace), *T*_2_ (middle of the furnace), and *T*_3_ (inlet of furnace) respectively. The flow rates of silane are denoted as *F*_1_ (bottom of the furnace) and *F*_2_ (inlet of furnace), while the pressure is set at 0.3 Torr. Curve 1 (*T*_1_ = 640 °C, *T*_2_ = 640 °C, *T*_1_ = 640 °C, *F*_1_ = 150 sccm, *F*_2_ = 150 sccm, *t* = 55 min). Curve 2 (*T*_1_ = 640 °C, *T*_2_ = 640 °C, *T*_1_ = 640 °C, *F*_1_ = 150 sccm, *F*_2_ = 180 sccm, *t* = 52 min). Curve 3 (*T*_1_ = 640 °C, *T*_2_ = 645 °C, *T*_1_ = 650 °C, *F*_1_ = 150 sccm, *F*_2_ = 180 sccm, *t* = 50 min). Arrange the substrates vertically within the quartz boat, ensuring a 1 cm gap between each individual substrate.

The significance of high-volume manufacturing in industrial production cannot be overstated. In the subsequent study, we delve into the processing technology pertaining to film thickness and uniformity in mass production. Polysilicon films vary in thickness and uniformity depending on the *T*_d_, deposition time and the flow rate of silane during deposition.^23^ As depicted in Curve 1, when *T*_1_, *T*_2_, and *T*_3_ are set to 640 °C and *F*_1_, *F*_2_ are adjusted to 150 sccm, the thickness of the polysilicon film near the inlet of furnace is comparatively minimal, indicating a pronounced disparity among the films. This may be related to the lower reaction temperature caused by the faster heat dissipation rate at the inlet of furnace. In order to minimize the variation in polysilicon film thickness across substrates, we augmented the *F*_2_ = 180 sccm, as illustrated in Fig. 7 Curve 2. The results demonstrate a significant reduction in the film thickness difference between the substrates, accompanied by an enhanced level of uniformity. Moreover, the temperatures were set as *T*_1_ = 640 °C, *T*_2_ = 645 °C, and *T*_3_ = 650 °C, while *F*_1_ = 150 sccm, *F*_2_ = 180 sccm, as shown in Curve 3. The difference in film thickness between the substrates is further reduced (<30 nm), and the uniformity is further improved (3%). By employing this procedure, a simultaneous deposition of polysilicon film on 100 substrates is achieved, with precise control over the thickness of the film through accurate regulation of deposition time.

**Fig. 8:**
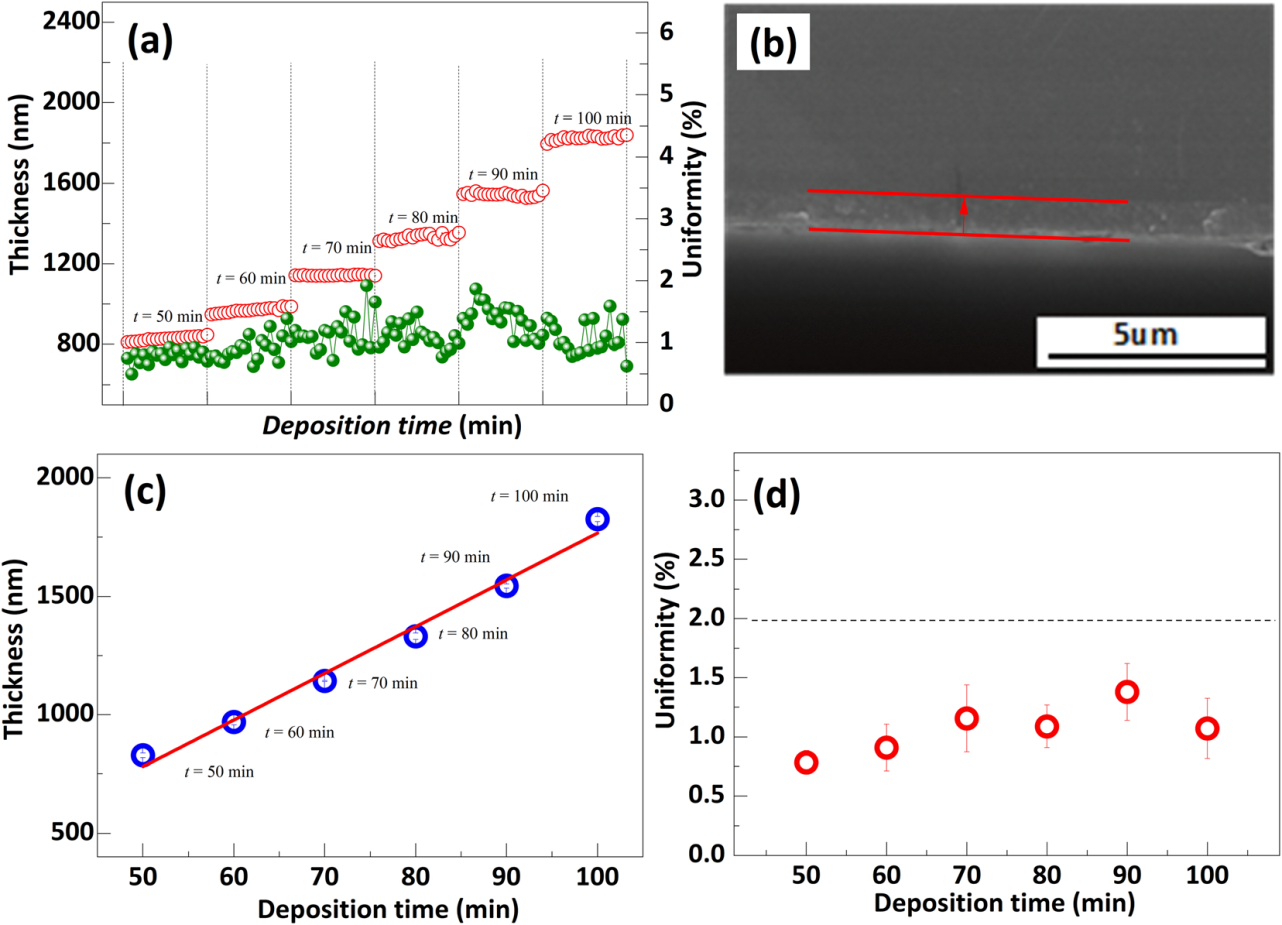
(a) Film thickness and uniformity of 20 substrates in the same deposition process. (b) SEM characterization of cross section of polysilicon film with thickness of 800 nm. The change of film thickness (c) and uniformity (d) as a function of deposition time. The deposition process is *T*_1_ = 640 °C, *T*_2_ = 645 °C, *T*_3_ = 650 °C, *F*_1_ = 150 sccm, *F*_2_ = 180 sccm, and the pressure is 0.3 Torr.

There is a strong correlation between the grain size and thickness of the polysilicon film as a depletion layer and the gettering effect. To investigate the gettering effect, the different thicknesses polysilicon film was deposited at variation time, illustrated in Fig. 8. Based on the results, the thickness of polysilicon film increases in proportion to the deposition time. Specifically, a film of polysilicon with a thickness of 1800 nm can be obtained after 100 min at a rate of 16 nm min^−1^, and its uniformity is satisfactory. In addition, based on the SEM cross-section images, the film thickness was observed to be approximately 800 nm, which agrees with the results of the film thickness measurement instrument when the deposition time is 50 min.

### 3.3 Effect of backside polysilicon film on Cu out-diffusion

**Fig. 9:**
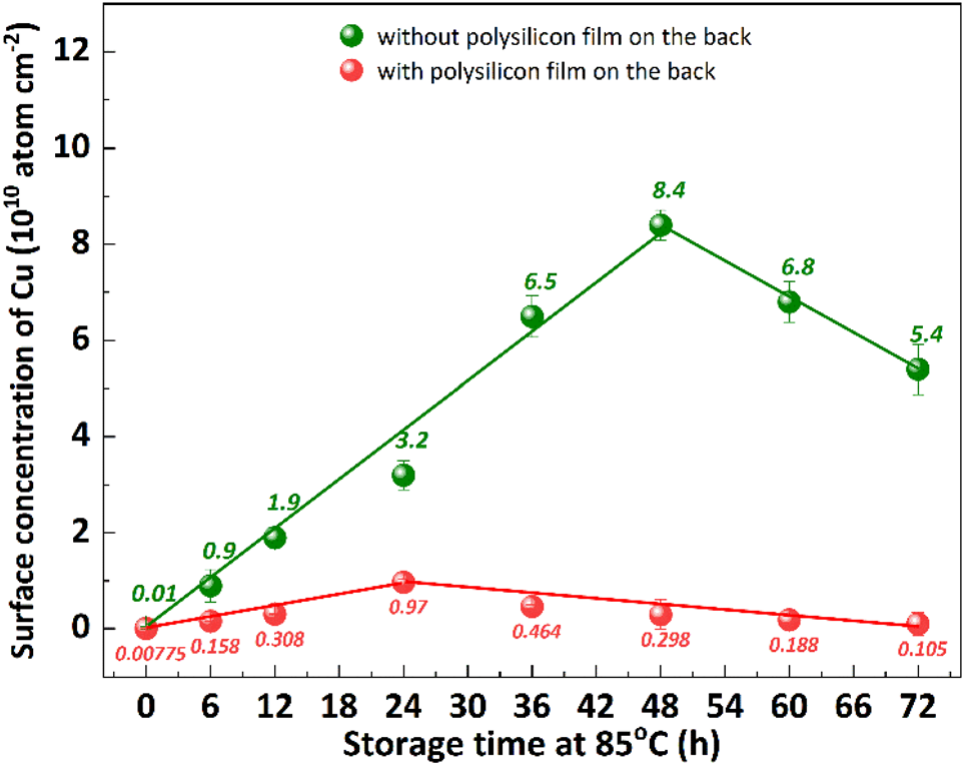
Effect of polysilicon film on Cu out-diffusion. All the substrates were contaminated with the same amount of Cu and divided into two groups. One group substrates grew polysilicon film through LPCVD and remove front side polysilicon film by polishing, and the other group did not do any treatment. After RCA cleaning, the two groups of substrates are simultaneously placed in an 85 °C environment for different time thermal treatment. The three temperature zones of the LPCVD furnace are set to *T*_1_ = 640 °C, *T*_2_ = 645 °C, *T*_3_ = 650 °C, silane flow *F*_1_ = 150 sccm and *F*_2_ = 180 sccm, and the pressure is 0.3 Torr.

Impurity gettering in silicon is an indispensable technology to avoid device degradation by heavy metal contamination.^28^ Polysilicon back seal, which refers to the deposition of thin polysilicon films on the back side of silicon substrates, is a commonly used out gettering technique.^11^ The previous studies have demonstrated that the implementation of a polysilicon film at the backside of p+ substrates effectively inhibits the diffusion of segregated metal from the bulk silicon to the epitaxial layer.^29^ This is attributed to the extremely low diffusivity of metal in polysilicon, making it an exceptional getter material.^30^ The impact of polysilicon film growth on the backside of the substrate on Cu out-diffusion at 85 °C was investigated, as shown in Fig. 9. The p+ silicon substrates were subjected to a 20 minutes contamination process with CuCl_2_ and subsequently cleaned using RCA cleaning to ensure that the surface Cu concentration is below 0.02 × 10^10^ atom per cm^2^. After undergoing thermal treatment at 85 °C for 48 hours, the p+ substrates without a polysilicon film exhibit a surface Cu concentration of 8.4 × 10^10^ atom per cm^2^, indicating the bulk Cu out-diffusion to the front surface of p+ substrates. The Cu concentration on the front surface of the p+ substrate, with a polysilicon film grown on the back, exhibits an initial increase followed by a subsequent decrease, reaching a maximum value of 0.97 atom per cm^2^ after 24 hours of thermal treatment at 85 °C. The obtained result suggests that the presence of a polysilicon film on the backside of the substrate effectively inhibited bulk Cu out diffusion to front surface.

**Fig. 10:**
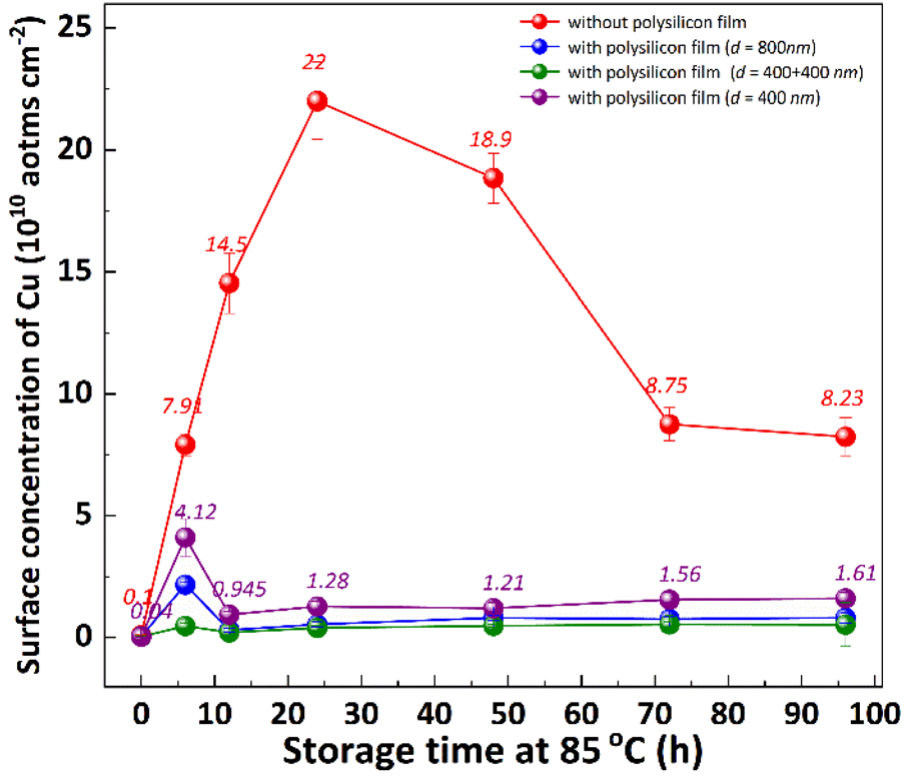
The effect of polysilicon film thickness and structure on Cu diffusion. The “without polysilicon film” represents the substrates without polysilicon film on the back of substrates. “with polysilicon film (*d* = 800 nm)” represents the substrates with polysilicon film about 800 nm thickness on the back of substrates. The “with polysilicon film (*d* = 400 nm + 400 nm)” represents the substrates with polysilicon film by two-step deposition about 800 nm thickness on the back of substrates. The “with polysilicon film (*d* = 400 nm)” represents the substrates with polysilicon film by deposition about 400 nm thickness on the back of substrates. The three temperature zones of the LPCVD furnace are set to *T*_1_ = 640 °C, *T*_2_ = 645 °C, *T*_3_ = 650 °C, silane flow *F*_1_ = 150 sccm and *F*_2_ = 180 sccm, and the pressure is 0.3 Torr, and the film thickness is controlled by the deposition time.

The multilayer structure of thin films is closely related to its mechanical properties,^31^ but there are few studies about its effect on the diffusion of Cu. To investigate the impact of polysilicon film thickness and structure on Cu out-diffusion, polysilicon films with thicknesses of 400 nm and 800 nm were deposited on the backside of the substrates using different deposition techniques, as shown in Fig. 10. The p+ silicon substrates were subjected to a 30 minutes contamination process with CuCl_2_ and subsequently cleaned using RCA cleaning to ensure that the surface copper concentration is below 0.02 × 10^10^ atom per cm^2^. The experimental results demonstrate that the Cu concentration on the front surface of the substrate exhibits an initial increase followed by a subsequent decrease during the heat treatment process, reaching its peak after 48 hours of thermal treatment. Similarly, for the substrate with a polycrystalline silicon film on the back, a comparable trend is observed, however, the maximum Cu concentration on the front surface is attained after 6 hours of thermal treatment. The maximum concentration of Cu on the front surface of the substrate, without a polysilicon film on the back, is 22.0 atom per cm^2^. When the 400 nm thick polysilicon film is deposited on the back, the maximum Cu concentration on the front surface of the substrate decreases to 4.1 atom per cm^2^. Increasing the thickness of the polysilicon film to 800 nm further reduces the maximum Cu concentration on the front surface of the substrate to 2.2 atom per cm^2^. When growing an 800 nm thick polysilicon film in multiple steps on the back, it significantly lowers down and reaches a minimum value of 0.47 atom per cm^2^. With the prolongation of thermal treatment duration, there was no significant increase observed in the Cu concentration on the substrate surface of the backside-grown polysilicon film. The results demonstrate that the growth of polysilicon film on the backside of substrates significantly inhibits the out diffusion of bulk Cu, with a more pronounced effect observed as the thickness of polysilicon film increases. Interestingly, an enhanced inhibition of Cu diffusion can also be achieved by increasing the number of layers through multiple depositions while keeping the total thickness of polysilicon film constant.

In mono-silicon, heavy metal impurities or micro-defects exhibit migration and resolidification at specific temperatures^32^ due to the interactions between impurities and defects.^33^ Exploiting this phenomenon, mechanical damage, defects, and polysilicon film are intentionally induced on the backside of the substrates to getter of heavy metal impurities from the device's active region to these specifically introduced areas.^4,30^ The getter of metals by polysilicon films can be elucidated through “segregation-induced gettering” mechanism.^34,35^ Polysilicon is composed of many single crystals with different orientations connected by grain boundaries. The atomic arrangement in the grain boundary layer is less compact than that in the crystal lattice, resulting in smaller distortions of metal impurities within this region. As a result, the distortion energy is significantly reduced, facilitating enhanced impurity absorption within the grain boundary layer.^33,36^ The backside gettering sites reduced the activation energy of the formation of metal silicides at a given temperature when the solubility limit has been reached.^34,37^ Consequently, thicker polysilicon films exhibit a higher density of grain boundaries and enhanced getter efficiency. The multilayer film structure formed by multistep depositions exhibits a higher density of inter-film interfaces compared to the monolayer film structure obtained through a one-step deposition, resulting in enhanced getter efficiency under the same film thickness.

## 4. Conclusion

The surface Cu concentration of the p+ monosilicon substrate, which has been quantitatively contaminated, exhibits an initial increase followed by a subsequent decrease with the increase of storage time. It is noteworthy that the highest surface Cu concentration is observed after 6 months of storage at 25 °C, while a similar peak is reached after 48 hours of storage at 85 °C. The bulk Cu can undergo diffusion on the substrate surface through out-diffusion for a duration of 48 hours at a temperature of 85 °C, while surface Cu can be effectively eliminated by RCA cleaning. Consequently, the repeated application of both out-diffusion and RCA cleaning processes enables efficient elimination of bulk Cu contamination. The out-diffusion of Cu can be effectively prevented by the growth of a polysilicon film on the back of the substrate, due to enhanced Cu precipitation facilitated by grain boundaries and disordered structure within the polysilicon film. Moreover, increasing temperature and reducing grain size can eliminated internal residual stress in LPCVD-deposited polysilicon films.

## Data availability

The data included in this paper can be provided on request.

## Conflicts of interest

There is no conflicts to declare.

## Supplementary Material
